# Palliative care need and management in the acute hospital setting: a census of one New Zealand Hospital

**DOI:** 10.1186/1472-684X-12-15

**Published:** 2013-03-28

**Authors:** Merryn Gott, Rosemary Frey, Deborah Raphael, Anne O’Callaghan, Jackie Robinson, Michal Boyd

**Affiliations:** 1 Faculty of Medical and Health Sciences, University of Auckland, Building 505, 85 Park Road, Grafton, New Zealand; 2 Auckland District Health Board, Auckland, New Zealand; 3 Waitemata District Health Board, Westlake, New Zealand

**Keywords:** Palliative care, End of life, Census, Healthcare, Acute, Hospital

## Abstract

**Background:**

Improving palliative care management in acute hospital settings has been identified as a priority internationally. The aim of this study was to establish the proportion of inpatients within one acute hospital in New Zealand who meet prognostic criteria for palliative care need and explore key aspects of their management.

**Methods:**

A prospective survey of adult hospital inpatients (n = 501) was undertaken. Case notes were examined for evidence that the patient might be in their last year of life according to Gold Standards Framework (GSF) prognostic indicator criteria. For patients who met GSF criteria, clinical and socio-demographic information were recorded.

**Results:**

Ninety-nine inpatients met GSF criteria, representing 19.8% of the total census population. The patients’ average age was 70 years; 47% had a primary diagnosis of cancer. Two thirds had died within 6 months of their admission. Seventy-eight of the 99 cases demonstrated evidence that a palliative approach to care had been adopted; however documentation of discussion about goals of care was very limited and only one patient had evidence of an advance care plan.

**Conclusion:**

One fifth of hospital inpatients met criteria for palliative care need, the majority of whom were aged >70 years. Whilst over three quarters were concluded to be receiving care in line with a palliative care approach, very little documented evidence of discussion with patients and families regarding end of life issues was evident. Future research needs to explore how best to support ‘generalist’ palliative care providers in initiating, and appropriately recording, such discussions.

## Background

In most developed countries, the majority of people spend time in acute hospitals during the last year of life [1,2] and a significant proportion die in this setting [3,4]. Studies conducted in Australia, the UK and Belgium have concluded that, at any one time, 13-36% of inpatients meet criteria for palliative care need [5-8]. Increased recognition of the important role played by hospitals in palliative care management has prompted demand for more evidence to inform service improvements [9]. In particular, a need to define the palliative care inpatient population and gain further understanding of the care and treatment these patients receive has been identified [10].

Only a minority of inpatients with palliative care need will receive specialist palliative care input [6-8] with the majority receiving care from ‘generalist’ or ‘primary’ palliative care providers who have not received postgraduate education and training specific to palliative care management (Frey et al., under review) [10-12]. Policy in a number of countries recommends these clinicians adopt a palliative approach to care with patients with life limiting illness [13-15], defined in New Zealand as an ‘open attitude toward death and dying by all service providers working with patients and their families, [which] respects the wishes of patients in relation to their treatment and care’ [16]. However, there is evidence from a number of countries that such an approach is not routine within acute hospital settings. For example, recent research conducted in two acute hospitals in England demonstrated that UK guidelines, which recommend a structured ‘transition’ to a palliative approach to care amongst hospital inpatients likely to be in the last year of their life [17], are very far from being implemented in routine practice, thereby denying many patients a choice to be involved in end of life decision-making [18]. A study of seriously ill hospitalised patients by Teno et al. [19] found that medical care was inconsistent with treatment goals for more than one third of those patients who preferred a palliative rather than life prolonging approach to care. Two recent Australian studies have identified a need to improve the diagnosis of dying and optimise the delivery of palliative care in hospital settings [7,20]. It is within this context that we identified an urgent need to build the research evidence base regarding palliative care management within acute hospitals in New Zealand.

### Research aim

To establish the numbers and characteristics of hospital inpatients who meet prognostic criteria indicating palliative care need and describe the extent to which their care and treatment is in line with a palliative approach to care.

### Research objectives

This research sought to determine both the *proportion* of hospital inpatients meeting criteria indicating they were likely to be in the last year of life as well as the *characteristics* of patients identified as being likely to be in the last year of life. Finally, the research determined whether there was evidence that a *palliative approach to care* had been adopted for this patient group.

## Methods

### Design and rationale

This study comprises one phase of a larger research project which aimed to explore key aspects of palliative care management in acute hospitals in New Zealand, using Auckland District Health Board (ADHB) as a case study. ADHB, the largest DHB in New Zealand (in terms of budget), was selected for inclusion in the study due to: 1) a higher than average percentage of hospital deaths compared with other DHBs (approx. 50%); [9] 2) a culturally and ethnically diverse patient population; and 3) an on-going commitment to change practice relating to palliative care. Approval for the study was granted by the hospital research ethics committee and the regional ethics committee (NTX/10/EXP/144).

### Instrument and procedure

Patients were included in the study if they met one or more of the Gold Standards Framework (GSF) clinical prognostic indicators for palliative care need [21]. The GSF prognostic indicator guide was developed in the UK to help clinicians identify which patients are likely to be in the final 12 months of life and might be in need of palliative care. The census data collection instrument, which incorporated the GSF prognostic indicator guide, was developed for use in the UK [8,22] and adapted to the New Zealand context (e.g. use of relevant socio-demographic categories and medical acronyms appropriate to the study location) in collaboration with two experienced palliative care clinicians from the Hospital Palliative Care Team, a Palliative Medicine Consultant (AOC) and a Palliative Care Nurse Practitioner (JR), who also undertook all data collection. Wards were surveyed sequentially between 2 May and 17 June 2011 with data collection for each ward completed over no more than a one day time period. This method provided a ‘snapshot’ of the cases present in a single point in time. Patients were included if they were over18 years and resident on the ward at 9 am on the day the ward was surveyed [8,22]. Critical care, cardio-thoracic intensive care, maternity, emergency department (including the assessment and planning unit) and paediatrics were excluded.

AOC and JR extracted relevant data from the medical records of eligible inpatients. For patients who met GSF criteria, supplementary information was gathered from patient notes and outpatient letters (see Additional file 1). Patients were screened using indicators that might suggest they were being managed in line with a palliative approach. These indicators were adopted from the UK study [8,23] and modified to the NZ context. These indicators comprised: evidence of an ACP, being on the Liverpool Care of the Dying Pathway, Referral to the Hospital Palliative Care Service, appropriate prescription of opioids, use of syringe driver for symptom control, presence of palliative care alert and documentation of a Palliative approach to resuscitation status in the case of respiratory or cardiac arrest.

A second step was taken to assess whether the management of the patient was appropriate for the individual patient within a palliative care context. In the absence of validated criteria, and given the importance of considering individual circumstances when making this determination, context specific expert clinical judgment (by AOC and JR) was deemed the most suitable approach. Several factors were taken into account including code status, whether the level of investigations was appropriate given the extent of the disease as well as evidence of effective symptom management. Effective symptom management was defined as documented evidence that pain and other symptoms (e.g. dyspnoea, fatigue and depression) had been recognised, diagnosed and treated. Written documentation of whether discussions had been held with patients and families in relation to prognosis and goals of care was considered. In addition an assessment was made based on the evidence as to whether limitations of treatment would have been clear to an out of hours on call clinician. Documentation of a referral to hospital palliative care team if symptom management was ineffective was also included in the assessment. All of the above evidence was considered in conjunction with the presence of one or more indicators of the adoption of a palliative approach to assess the appropriateness of care delivered by the care team.

### Analysis

Data were cleaned and coded into an SPSS database. Pearson’s chi square test was utilized to detect significant differences between groups for data collected at the nominal or ordinal level. Interval-level data were analysed utilising *t*-tests, Pearson correlations and one-way ANOVA.

## Results

Ninety-nine (19.8%) of the inpatients included in the census (n = 501) met at least one of the GSF prognostic indicators, suggesting that they were likely to be in the last year of life. The following results relate to these 99 patients.

### Socio-demographic characteristics of patients

Approximately half of the patients were female (n = 50; 51%) and the mean age for the sample was 70 years, with the greatest proportion aged over 83 years (Table 1). The majority co-habited (n = 62; 62%), 16 lived in Aged Residential Care (16%), and 14 lived alone (14%). Most patients were New Zealand European (n = 64; 64%), with seven (7%) Māori, four (4%) Chinese, four (4%) Samoan, three (3%) Tongan and two (2%) Niuean. The majority of patients resided in the Auckland region, with smaller numbers resident in other areas of New Zealand (Table 1).

**Table 1:** Baseline characteristics of patients with palliative care needs

	**Age group**					
**Variable**	**Lowest −49**	**50-60**	**61-71**	**72-82**	**83 and older**	***Total***
**Gender**						
Male	4	9	9	13	12	47
Female	5	8	11	9	17	50
Transgender	1	0	0	0	0	1
**Residence**						
Co-Habited	9	16	13	14	10	62
Lived Alone	1	1	3	3	6	14
Nursing Home/Residential Care	0	0	1	2	13	16
Unknown	1	0	0	3	0	4
**District Health Board of Residence**						
Auckland	7	7	9	16	27	66
Counties Manukau	3	2	3	3	0	11
Waitemata	1	3	6	1	2	13
Northland	0	3	2	2	0	7
Bay of Plenty	0	1	0	0	0	1
**Ethnicity**						
New Zealand European	5	8	12	13	26	64
Māori,	1	4	2	0	0	7
Samoan	0	0	2	2	0	4
Tongan	2	1	0	0	0	3
Niuean	1	0	0	1	0	2
Indian	0	0	0	1	0	1
Chinese	0	0	2	1	1	4
Other	1	3	1	4	2	11

### Diagnostic information

Of the ninety-nine patients who met GSF criteria, almost half (n = 46; 46.5%) had a primary diagnosis of cancer. The most common non-cancer primary diagnosis was heart disease (n = 11; 11.1%), followed by renal disease (n = 8; 8.1%) \ chronic obstructive pulmonary disease \ (n = 5; 5.1%), and frailty (n =5; 5.1%).

Nineteen patients had at least one recorded co-morbid condition, 24 had two co-morbid conditions, and 18 had three or more co-morbid conditions. The most common co-morbid conditions reported were heart problems (heart failure, heart disease, angina) (n = 37; 37.4%), chronic renal disease (n = 16; 16.2%), diabetes (n = 15; 15.2%), cancer (n = 12; 12.1%), respiratory disease (COPD, asthma) (n = 12; 12.1%) and stroke (n = 10; 10.1%). Patients over 65 years were more likely to have heart problems (heart failure, heart disease, and angina) as a co-morbid condition than patients less than 65 years (*χ*^2^ (1, n = 99) = 7.92, *p* < .01). 29% of patients had recorded evidence of cognitive impairment, including 4% with a recorded diagnosis of dementia; patients over 65 years were more likely to have cognitive impairment (*χ*^2^ (1, n = 99) = 5.61, *p* < .01) than younger patients. 7% of patients had English recorded as a second language, 4% lacked ability to communicate for other reasons and 2% had learning difficulties.

### Hospital admissions information

Half of patients were admitted to hospital from the Emergency Department (ED) (n = 48; 50%), and 20% from the Admissions and Planning Unit (APU) (n = 20; 21%) which accepts GP referrals for patients thought to require hospital admission. Patients with a major diagnosis other than cancer (*χ*^2^ (1, n = 86) = 9.08, *p* = .003) and patients > 65 years of age were more likely to be admitted via ED *χ*^2^ (1, n = 96) = 6.0, *p* = .014). Patients who were not referred to the palliative care team during the census admission (n = 37) were also more likely to have been admitted through the ED than those patients with a referral to the palliative care team who were admitted through ED (11) (*χ*^2^ (1, n = 92) = 4.29, *p* = .03).

Patients had an average of three admissions (M =3.03, SD = 2.0) over the previous year, with an average of 33 days (M = 33.1, SD = 29.9) spent in hospital over the previous 12 months. There was a moderate negative relationship between number of hospital admissions and age, (*r* (94) = −.30, *p* = .002), indicating that the number of hospital admissions decreased with the age of the patient. There was also a significant effect (*p* < .01), of ethnicity on number of hospital admissions in the last twelve months (F (3, 89) = 5.92, p = .001). Post hoc comparisons using the Tamhane’s test indicated that the mean number of admissions over 12 months for Pacific Peoples (M = 5.07, SD = 2.67) was significantly higher than for NZ European (M = 2.66, SD = 1.62) or ‘other’ ethnic groups (M = 2.88, SD = 2.80) patients.

### Indicators of a palliative approach

Overall, 78.8% (n = 78) of patients had at least one indicator that suggested they were being managed in line with a palliative approach. Of these, 23 patients met just one indicator, 32 patients met two indicators and six patients met three indicators of adoption of a palliative care approach. Fifty-six (56%) had documentation of a palliative approach with regard to resuscitation status for respiratory arrest. Seventeen (17.2%) had documentation of a palliative approach to resuscitation status for cardiac arrest. Patients *under* the age of 65 years (n = 37) more likely to have evidence of a palliative approach to resuscitation status for cardiac arrest compared with patients over the age of 65 years (n = 52) (*χ*^2^ (1, n = 87) = 7.28, *p* < .05). Only one patient (1%) had a documented Advance Care Plan (ACP) (see Table 2).

**Table 2:** Indicators for the adoption of a palliative care approach (n = 99)

***Indicators of palliative approach***	***Frequency***	***%***	***Missing data***
Evidence of ACP	**1**	**1.0**	**7**
Placed on Liverpool Care Pathway	**9**	**9.1**	**7**
Referred to Hospital Palliative Care	**32**	**32.3**	**4**
Prescription of repeated long term opiates	**20**	**20.2**	**7**
Use of a syringe driver	**10**	**10.1**	**7**
Palliative care alert hospital	**11**	**11.1**	**7**
Palliative care alert hospice	**15**	**15.2**	**8**
Resuscitation status (respiratory)	**56**	**56.6**	**12**
Resuscitation status (cardiac)	**17**	**17.2**	**10**

### Appropriateness of approach to care

As outlined in Table 3, for the majority of patients (93%) the level of investigation as documented in the case notes was deemed appropriate given the extent of the disease. 88.9% demonstrated evidence of effective symptom management.

**Table 3:** Quantitative indicators of appropriateness of approach to care (n = 99)

***Indicators***	***Frequency***	***%***	***Missing data***
Code status appropriate?	**76**	**76.8**	**1**
Is the level of investigation appropriate?	**93**	**93.9**	**1**
Effective symptom management?	**88**	**88.9**	**1**
Family/patient information needs ascertained?	**31**	**31.3**	**2**
Goals of care clearly documented?	**29**	**29.3**	**1**
Would limitations of treatment be clear to an on call out-of-hours clinician (medical care provided outside normal GP surgery hours)?	**29**	**29.3**	**1**
Involvement of Pacific Family Support Kai Atawhai (Non NZ European patients) (n = 24)	**2**	**8.3**	**2**

Problems in terms of communication were also evident in the notes. Goals of care were documented clearly for only 29% of patients. Furthermore, written evidence that patient and family information needs had been ascertained was present for 31% of patients.

There were 19 cases where there was no evidence that the management of the patient was appropriate within a palliative care context. In these instances care was focused primarily on the acute presentation with little or no evidence that the goals of care had been considered. Examples of representative cases are provided in Table 4.

**Table 4:** Evidence of an appropriate approach to care: examples

***Approach Appropriate?***	***Frequency***	***Concept***	***Frequency***	***Illustrative quote***
Yes	43	*However -.ACP/discussion around future goals would be useful*	18	*‘Appropriate for management of CHF but clear that this is becoming more difficult with worsening renal function and decreased function and more frailty. ACP discussion and goals of care required.’*
		*Appropriate Care*	21	*‘Approach appropriate for management of sepsis. Code status appropriate given age and co-morbidities.’*
		*New Diagnosis*	5	*‘Appropriate management, given new diagnosis. Most of this admission has been focused on obtaining diagnosis, deciding on a management plan whilst managing symptoms.’*
No	19	*No Discussion*	14	*‘No discussion with family regarding goals of care and the role of palliative care alongside treatment of chest infection. Admission only 24 hours if he does not respond to IV antibiotics, further family discussion is required.’*
		*Approach to Management not Appropriate*	5	*‘Appropriate management of sepsis on admission on the 09/05/11 but failed to improve and began to deteriorate from cardiac point of view with tachy-brady syndrome and sepsis. Code red called 26/05/11 when decision made not to escalate treatment. Family seem distressed by changes in the focus of care.’*

### Hospital palliative care service referral

The hospital palliative care service provides consultative patient-focused specialist palliative care, in conjunction with the primary multi-disciplinary team on the ward. Referral criteria are based on the Leeds Palliative Care Referral Criteria [23] and referrals are accepted from any health professional caring for hospital inpatients provided that the medical team is aware of and has agreed to the referral being made. 32.3% of the 99 patients who met GSF criteria had already been referred to the hospital palliative care service (see Table 3), two thirds of whom had a major diagnosis of cancer (n = 22). Patients referred to the palliative care service were more likely to reside in district health boards outside of Auckland (n =19) in comparison to those who resided within Auckland District Health Board (n =13) (*χ*2 (1, n = 94) = 16.83, p = .000) and more patients with documented goals of care prior to the referral (n = 22) had been referred to the hospital palliative care team than those patients without documented goals of care (n = 9) (*χ*2 (1, n = 94) = 34.89, p < .001). 26.2% of patients had a palliative care alert, meaning that they had had previous contact with a specialist palliative care service in the hospital or community.

## Discussion

This census of palliative care need and management in one acute hospital in New Zealand confirms this setting as a significant site of palliative and end of life management. One fifth of inpatients met GSF prognostic indicator criteria for palliative care need,. Comparison across studies is complicated by a lack of consistency in definitions of the palliative care inpatient population. Our finding is consistent with a UK study where medical and nursing staff were asked to identify patients who met a standardised definition of palliative care need [18], although it is higher than a number of earlier studies [5,24,25], which is likely to reflect the recent expansion of the definition of palliative care as involving more than terminal care [26]. Two recent studies [7,8] have reported a prevalence of palliative care need of 35% and 36%, but marked methodological differences preclude direct comparisons. To the best of our knowledge our study is the only conducted to date using a standardised tool with a total hospital inpatient population to identify patients likely to be in the last year of life on the basis of predetermined diagnostic and prognostic criteria.

Patients meeting criteria for palliative care need had an average age of 70 years, with the greatest proportion aged more than 83 years. This is unsurprising given that deaths from chronic disease disproportionately affect older people; a recent New Zealand palliative care needs assessment estimated that 78% of people dying with palliative care needs are aged over 65 years, with approximately one quarter aged over 85 years [13]. Findings also correspond with previous research [8,18,27] and confirm the important role played by the acute hospital in determining the end of life experience of older people. The finding that older patients were less likely than younger patients to have evidence of a palliative approach to resuscitation status for cardiac arrest recorded in their notes runs counter to most, but not all, of the published evidence [28]. In the case of the present study the results may be better explained by the culture of the medical specialty associated with particular wards rather than by age of the patients present. Research by Kaufman [29] indicates that the decision to start or continue life-extending interventions (e.g. dialysis) in an elderly population (70 yrs. and over) is largely driven by institutional culture, which will vary between medical specialties. However, insufficient numbers mean that such associations cannot be determined on the basis of our data and further research is needed to explore these possible explanations.

Almost half of our patient sample had a primary diagnosis of cancer. This was expected given that cancer continues to be the leading cause of death in New Zealand (29.4%) [30] and that the hospital provides the Regional Cancer Service. However, in line with previous studies [6,27], a similar proportion of patients had a primary diagnosis other than cancer, including one third of patients referred to the hospital palliative care team. National figures from a Health Needs Assessment in New Zealand [31] indicated differences in the proportion of cancer (average 79%) and non-cancer patients (average 21%) receiving care from hospice across the country. The proportion of non-cancer referrals found in the present study is considerably higher than that recorded for hospice referrals in the New Zealand Health Needs Assessment or in the UK [31,32]. It is also higher than the proportion of non-cancer referrals to specialist palliative care services reported in a study of three Australian hospitals [7]. This finding is encouraging in light of the New Zealand goal of providing specialist palliative care on the basis of need, not diagnosis [33].

Half of patients presented through the Emergency Department (50%) and, interestingly, patients aged over 65 years, and patients with conditions other than cancer, were more likely to access the hospital via this route. This is likely to reflect the fact that patients with cancer are usually admitted through the oncology day stay unit. Previous research, albeit limited, supports the conclusion that people are high users of urgent care services during their last year of life. For example, Rosenwax et al. [1], reported that 70% of a cohort of 1071 people in Western Australia who died of a condition amenable to palliative care intervention experienced at least one emergency presentation during their last year of life. Whilst high use of urgent care services is likely to partly reflect gaps in community palliative care provision, research in this area is limited [34].

The study also indicated a higher number of hospital admissions for Pacific patients in comparison to NZ European or ‘other” ethnic groups. This finding supports previous research by a 2004 jointly funded review by the Ministry of Health and the Ministry of Pacific Island Affairs, which concluded that Pacific peoples experience a higher rate of hospitalisations due to a number of barriers to access to primary health care, including both financial and cultural impediments [35].

### Palliative care approach

Whilst clinically the majority of patients in our study were considered to be managed appropriately given their illness stage, there was little recorded evidence of discussion of goals of care. Whilst this finding is not consistent with a study conducted in New Zealand which concluded that 82% of hospital decedents of all causes had a documented end of life discussion in their notes [36], it is in line with previous research confirming the limited extent of recording of such discussions during hospital admissions in the 12 months prior to death [37,38]. Moreover, it confirms that in New Zealand, in line with most countries for which evidence is currently available, levels of Advance Care Planning remain low, particularly for people with conditions other than cancer, and that prognosis is often rarely explicitly disclosed [12,39-41]. Levels of documentation of conversations relating to end of life care have also previously been reported to be poor; [42] improving documentation of end of life discussions is imperative if continuity of care is to be achieved and patient’s end of life preferences respected.

For those patients whose approach to care was not felt to be appropriate to their stage of illness, reasons included a focus on addressing the acute problem, rather than looking at the wider context of the patient’s illness trajectory. This finding is supported by earlier research in the US by Lynn and Goldstein [43], p. 812 who observed that: “patients with eventually fatal illnesses often receive routine treatments in response to health problems rather than treatments arising from planning that incorporates the patient's situation and preferences”. The opportunity provided by a hospitalisation to reflect on goals of care with the patient and their family and within the context of the overall illness trajectory is therefore missed. This finding lends weight to the call for the development of new evidence based interventions to support hospital clinicians in discussing goals of care with patients and their families [18].

### Limitations

This paper provides the first published data regarding the nature of the palliative care inpatient population within New Zealand and the care they receive, and to the best of our knowledge, is the only study conducted to date internationally to use a standardised tool to identify patients likely to be in the last year of life within a total hospital inpatient population. However, certain limitations must be acknowledged. Case note reviews of individual patients only indicate palliative care need based on diagnoses and cannot provide an individual assessment of need at the patient level. Secondly, clinical judgments were used to determine whether a palliative approach had been used (albeit by highly experienced palliative care clinicians) which are, by their very nature, subjective. Furthermore, no information was collected from patients and their families and therefore their perspective on care and treatment was not available. Finally, recruitment for this study was derived from one acute hospital. Therefore the generalizability of the results should be treated with caution.

## Conclusion

In this New Zealand study of one acute hospital one fifth of hospital inpatients met criteria for palliative care need, the majority of whom were aged >70 years. Whilst over three quarters were concluded to be receiving care in line with a palliative care approach, very little documented evidence of discussion with patients and families regarding end of life issues was evident. Future research needs to explore how best to support ‘generalist’ palliative care providers in initiating, and appropriately recording, such discussions if people are to be adequately involved in making decisions about their end of life care and treatment.

## Competing interests

The authors declared that they have no competing interests.

## Authors’ contributions

All authors (MG, RF, DR, AO’C, JR, MB) were involved in the conception, design and implementation of the research. MG, RF, DR, AO’C, JR were involved in the data analysis and interpretation, and drafting of the paper. All authors were involved in the review and approval of the final article for publication.

## Pre-publication history

The pre-publication history for this paper can be accessed here:

http://www.biomedcentral.com/1472-684X/12/15/prepub

## Supplementary Material

Additional file 1 Collected data. Click here for file
