# Supplementary material for: Palliative care need and management in the acute hospital setting: a census of one New Zealand Hospital
Source: BMC Palliat Care. 2013 Mar 28;12:15. doi: 10.1186/1472-684X-12-15 (PMC3636052; doi:10.1186/1472-684X-12-15)
Supplement: Additional file 1 — Collected data. [file 1472-684X-12-15-S1.docx]

**Collected Data**

Diagnosis specific prognostic information according to GSF prognostic indicator criteria

Reason for Admission

Estimated date of discharge

Expected discharge destination

Clinician Admitting

Source of Admission

Underlying diagnosis relevant to presenting condition

Co-morbidities

Date of first diagnosis

Living arrangements

Area of residence

**Evidence of preventable admission**:

-GP contact

-hospice contact within 24 hours

-presented with symptoms manageable by generalist +/- specialist palliative care service

-discharge documentation palliative

-prognosis or goals of care

-repeated admission

Other issues: Cognitive impairment / Learning Diffs / Lack of Communication / English as a second language/other

**Evidence of a palliative approach**-

-code red (respiratory)

-code blue (cardiac)

-evidence of ACP

-Liverpool Care Pathway

-referred to hospital palliative care

-prescription of repeated long term opiates

-use of a syringe driver

-palliative care alert hospital

**Evidence of Team adopting palliative approach**

-code status appropriate to level of disease

-level of investigation appropriate to disease

-symptom management

-patient/families’ needs for information ascertained

-opportunity for patient/family to discuss prognosis and goals of care

-goals of care clearly documented

- Would limitations of treatment be clear to an on call out of hour’s clinician that the patient was not receiving curative treatment?

Age

Gender

Ethnic origin

Number of hospital admissions in the last 12 months

Number of days spent in hospital in last 12 months
